# Enhancing Epoxy Composite Performance with Carbon Nanofillers: A Solution for Moisture Resistance and Extended Durability in Wind Turbine Blade Structures

**DOI:** 10.3390/ma17020524

**Published:** 2024-01-22

**Authors:** Angelos Ntaflos, Georgios Foteinidis, Theodora Liangou, Elias Bilalis, Konstantinos Anyfantis, Nicholas Tsouvalis, Thomais Tyriakidi, Kosmas Tyriakidis, Nikolaos Tyriakidis, Alkiviadis S. Paipetis

**Affiliations:** 1 CSMLab, Department of Materials Science & Engineering, University of Ioannina, 45110 Ioannina, Greece; a.ntaflos@uoi.gr (A.N.); g.foteinidis@uoi.gr (G.F.); 2 Shipbuilding Technology Laboratory, Department of Naval Architecture & Marine Engineering, National Technical University of Athens, 15780 Zografos, Greece; doraliangou@yahoo.com (T.L.); ebilalis@mail.ntua.gr (E.B.); kanyf@naval.ntua.gr (K.A.); tsouv@mail.ntua.gr (N.T.); 3 B&T Composites, Agrokthma Florina AA 1834, 53100 Florina, Greece; thomai@btcomposites.gr (T.T.); kosmas@btcomposites.gr (K.T.); nikos@btcomposites.gr (N.T.)

**Keywords:** wind turbine blades, glass-fibre-reinforced plastics (GFRPs), graphene nanoplatelets (GNPs), filament winding, environmental aging

## Abstract

The increasing prominence of glass-fibre-reinforced plastics (GFRPs) in the wind energy industry, due to their exceptional combination of strength, low weight, and resistance to corrosion, makes them an ideal candidate for enhancing the performance and durability of wind turbine blades. The unique properties of GFRPs not only contribute to reduced energy costs through improved aerodynamic efficiency but also extend the operational lifespan of wind turbines. By modifying the epoxy resin with carbon nanofillers, an even higher degree of performance can be achieved. In this work, graphene nanoplatelet (GNP)-enhanced GFRPs are produced through industrial methods (filament winding) and coupons are extracted and tested for their mechanical performance after harsh environmental aging in high temperature and moisture. GNPs enhance the in-plane shear strength of GFRP by 200%, while reducing their water uptake by as much as 40%.

## 1. Introduction

The global shift from fossil fuels to renewable energy sources, primarily wind energy, represents a pivotal moment in the ongoing battle against climate change. This transition, driven by mounting environmental concerns and the recognition of finite fossil fuel resources, signifies a commitment to a more sustainable and cleaner future. Wind energy offers an abundant and renewable source of power, significantly reducing greenhouse gasses [1]. GFRP’s lightweight yet strong nature is pivotal in ensuring the blades’ efficient rotation and optimizing energy production [2]. Furthermore, its resistance to corrosion is vital for withstanding harsh outdoor conditions where wind turbines are typically situated [3].

Epoxy resins represent a class of exceptionally versatile polymers utilized across diverse high-performance industries owing to their exceptional amalgamation of mechanical strength, chemical stability, and physical properties. They exhibit a broad range of applications, serving as vital components for structural elements [4]. Their inherent compatibility with a wide array of reinforcing fibres, minimal shrinkage during the curing process, low mass, and cost-effectiveness makes epoxy resins and fibre-reinforced epoxy composites attractive alternatives to traditional materials [5]. Epoxy-based composites offer unique advantages, particularly in high-stress settings characterized by exposure to moisture and elevated temperatures known to expedite degradation processes, ultimately leading to premature component failure. The presence of moisture initiates various adverse effects, such as swelling, plasticization, and overall material degradation. The water molecules permeate the epoxy resin matrix, effectively diminishing its mechanical properties through the establishment of hydrogen bonds with the hydrophilic functional groups within the epoxy structure. This interaction triggers a swelling effect, infiltrating the material’s “free volume” and substantially compromising its long-term durability [6].

The Integration of carbon nanofillers into epoxy composites has gained substantial attention within the realm of scientific investigation, primarily due to the immense potential for advanced applications [7]. These advanced epoxy/carbon nanocomposites are promising for mitigating the adverse impact of moisture, simultaneously enhancing their mechanical properties [8], and thus extending their operational lifespan. Extensive research efforts into exploring the influence of these diverse carbon fillers on the moisture absorption characteristics of epoxy resins have revealed notable enhancements in the resilience of the matrix when subjected to environmental exposure [9]. This improvement is intricately linked to the geometry and dimensions of the incorporated nanofillers [10]. These findings underscore the promising potential of carbon nanofillers in fortifying epoxy resins against moisture-induced degradation and advancing their overall performance characteristics, fostering their suitability for a range of advanced applications [11].

Graphene nanoplatelets (GNPs) are commonly commended for their ability to enhance the barrier properties of epoxy composites [12]. GNPs are stacks of graphene sheets with thicknesses from a few nm up to 100 nm [13]. The advantages of GNPs are their low manufacturing cost and capabilities for mass production. These characteristics make GNPs ideal for industrial applications. Enhancing epoxy GFRP with GNPs can result in epoxy nanocomposites with improved barrier, electrical, and mechanical properties. When a network of GNPs is formed in the matrix, it can significantly decrease the permeation of erosive substances like moisture by creating a tortuous path, forcing the water molecules to follow a complicated pathway [14]. Research has established a correlation between the geometry of GNPs, including high specific surface area and aspect ratio, and their efficacy as barriers [15].

In this study, physical and mechanical characterization through international standards was performed in pristine and aged unmodified (neat) and GNP-reinforced GFRP post environmental degradation in harsh environments of elevated moisture and temperature. The GFRPs were produced in an industrial environment using filament winding, which is deemed a sustainable manufacturing methodology for large structures such as wind turbine blades. For better evaluation of the results, dynamic mechanical analysis was performed to examine the effect of water ingress on the properties of the GFRP. This work encompasses all evaluation methodologies, including industrial manufacturing, to prove the viability of the nanomodification in the relevant industrial environment with the direct transfer beneficial effect of the nanomodification on real structures.

## 2. Materials and Methods

### 2.1. Materials

XGNPs C-300 graphene nanoplatelets, provided by XGSciences, Lansing, MI, USA, were used as the carbon nanofiller of choice for the improvement of the GFRP. The GNPs had a thickness of a few nanometres, lateral size smaller than 2 μm, and 300 g/m^2^ surface area with a Raman ID/IG ratio of 0.85 [16,17,18]. A commercial-grade epoxy resin, diglycidyl ether of bisphenol A (DGEBA) Epikote 828, was provided by Hexion, Columbus, OH, USA, along with complementary Epikure curing agent 866 and Epicure Catalyst 101 in a 10:8.3:0.15 mixing ratio. The epoxy viscosity at room temperature was 10.000 mPa s. E6-CR 386T by Jushi E-glass fibres designed for filament winding applications were applied for the manufacturing of the GFRP structures.

### 2.2. Dispersion and Manufacturing

High shear mixing was selected for dispersing the nanofillers in the polymer matrix. The dispersion protocol was performed using a laboratory dissolver device (Dispermat AE by Gentzman, Reichshof, Germany) supplied with a double wall vacuum container in combination with a thermostatic bath by GRANT capable of temperature control within ±1 °C accuracy. The conditions of the dispersion protocols were rotary speed of 3000 rounds per minute (rpm) and temperature of 25 °C. The selected nanofiller weight content was selected in a previously unpublished work to be 1% wt. GNP. Introducing 1% wt. GNP in the epoxy system showcased the optimum overall performance, including reduced water absorption and increased mechanical properties, in lab-scale manufactured GFRP.

**Figure 1:**
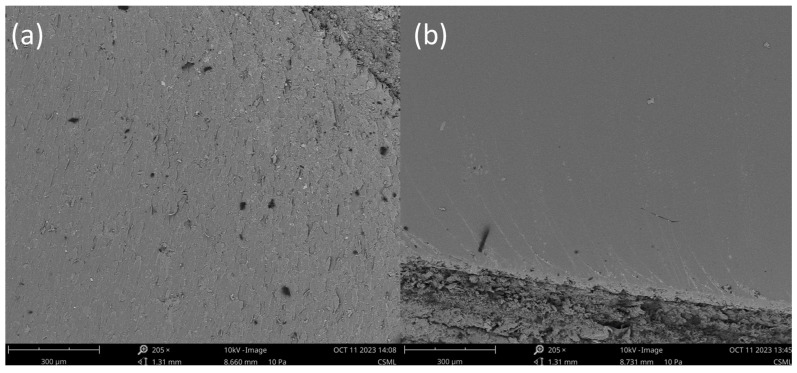
SEM images of (**a**) GNP-enhanced epoxy resin and (**b**) Neat epoxy resin post single-edged notched beam testing.

SEM spectroscopy was performed on a Phenom Pharos Desktop SEM by Thermo Fisher Scientific, Waltham, MA, USA. Epoxy matrix specimens were tested to evaluate the dispersion state. In Figure 1, two SEM images are presented: Figure 1a corresponds to the GNP-enhanced matrix specimen post single-edged notched beam (SENB) testing, while Figure 1b corresponds to the neat matrix post SENB. After examination, the dispersion of GNPs in the matrix was homogonous, while the fracture mechanisms presented were in line with the literature. GNPs introduced additional fracture mechanisms, increasing the fracture surface roughness [19].

**Figure 2:**
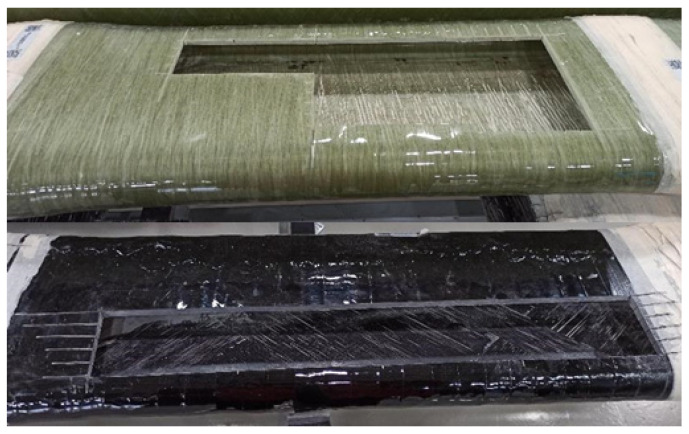
Filament wound GFRP structures for material mechanical characterization (neat resin above, modified resin below).

B&T Composites in Florina, Greece, produced two sets of large-scale industrial GFRP structures purely via filament winding (Figure 2), one set utilized a conventional resin, while the other employed resin modified with 1% wt. GNPs. Each set consisted of three composite variants with varying fibre orientations: 0°, 90°, and a biaxial orientation of approximately ±45°.

**Figure 3:**
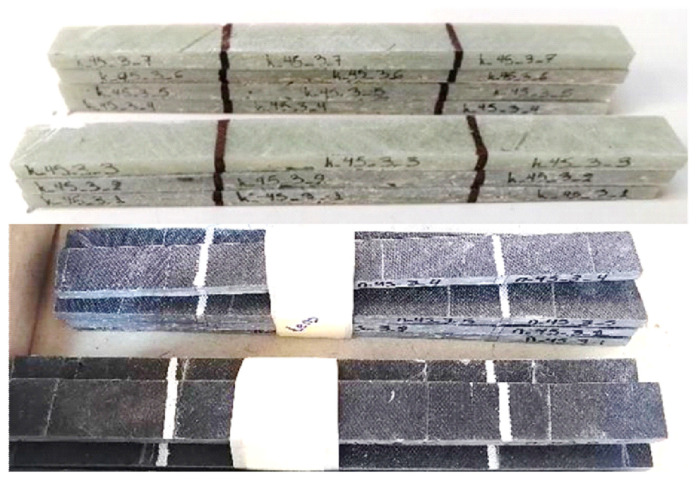
Typical GFRP coupons (neat resin above, modified resin below).

Coupons from the configurations above were collected and subjected to physical characterization based on the ISO 1172:1996 [20] standard to assess fibre content (calcination Method A) and ISO 1183-1:2004 [21] standard to determine coupon density (Method A, immersion method). The coupons’ dimensions were defined according to the ASTM D3039 [22] and ASTM D3518 [23] standards, used for the measurement of the tensile and the in-plane shear properties, respectively. Both types of coupons were plane and orthogonal, having length equal to 160 mm, width equal to 15.3 mm with coefficient of variation (CoV) = 1.8% for the neat resin coupons and equal to 15.7 mm with CoV = 2.3% for the modified resin ones, and thickness equal to 8.8 mm with CoV = 5.0% for the neat resin coupons and equal to 9.0 mm with CoV = 5.6% for the modified resin ones. Figure 3 depicts typical GFRP coupons. Both types of coupons were plane and orthogonal, having length equal to 160 mm, width equal to 15.3 mm with coefficient of variation (CoV) = 1.8% for the neat resin coupons and equal to 15.7 mm with CoV = 2.3% for the modified resin ones, and thickness equal to 8.8 mm with CoV = 5.0% for the neat resin coupons and equal to 9.0 mm with CoV = 5.6% for the modified resin ones.

Subsequently, these coupons were categorized into six groups as outlined in Table 1:

### 2.3. Mechanical Testing

**Figure 4:**
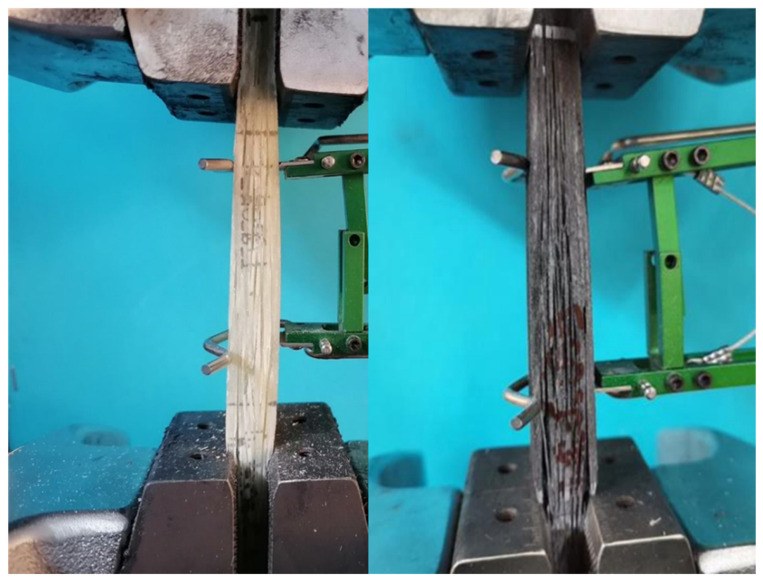
Tensile test of a 0° neat resin coupon (**left**) and of a 0° modified resin coupon (**right**).

Mechanical testing was carried out in a 250 kN capacity hydraulic testing machine. All tests were displacement controlled with an imposed displacement rate equal to 2 mm/min for the 0° coupons, 0.5 mm/min for the 90° coupons, and 1 mm/min for the ±45° ones. Strains were measured with the aid of an extensometer in the case of the tensile tests of 0° and 90° coupons (gauge length equal to 50 mm), and with the aid of a 5 mm gauge length, 0/90 strain gauge rosette, in the case of the ±45° coupons for measuring in-plane shear properties (Figure 4). The reaction force of the testing machine was also measured for each test, which, by dividing it by the respective cross section area of each coupon, resulted in the applied stress. Therefore, Young’s modulus and tensile strength were measured from the 0° and 90° coupons and shear modulus and shear strength from the ±45° ones.

### 2.4. Hydrothermal Aging

Hydrothermal exposure was performed on 5 coupons from each category of Table 1. The specimens were exposed to 70 °C temperature and 85% relative humidity in an environmental chamber. For all configurations, the coupons were sealed with commercial high-temperature-resistant silicon and aged for 90 days. The coupons were periodically weighed to determine their water absorption. Post degradation mechanical evaluation was performed.

### 2.5. Dynamic Mechanical Analysis

Dynamic mechanical analysis was performed in DMA Q850 (TA Instruments, New Castle, DE, USA) in 3-point bending configuration. The coupons tested were extracted from the 90° sample. The testing parameters were:

- Amplitude: 20.0 µm
- Frequency: 1.0 Hz
- Temperature scan: from 40 °C to 180 °C
- Heating rate: 3.0 °C/min

The DMA results were used to calculate the molecular weight between crosslinks according to the basic equation of rubber elasticity:

E_R_ = 3 (d/M_c_) RT (1)

where E_R_ is the storage modulus at the rubbery plateau, d is the density of the composite, R is the universal gas constant, and T is the temperature at the rubbery plateau. For this research the T at the rubbery plateau was set as Tg + 30 °C [24].

## 3. Results and Discussion

### 3.1. Water Uptake

**Figure 5:**
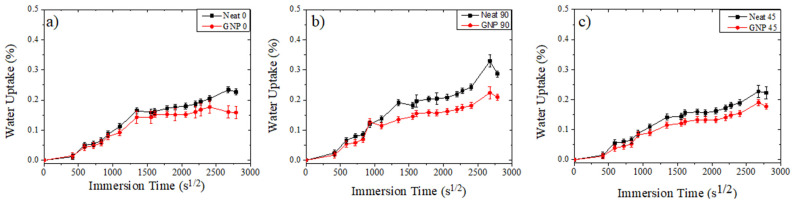
Average water uptake (%) per square root of time (s^1/2^) of neat and nano-modified (GNP) coupons. (**a**) 0 oriented coupons, (**b**) 90 oriented coupons, (**c**) 45 oriented coupons.

Before starting the moisture absorption tests, all coupons were dried in an oven at 60 °C until significant change in mass was not observed (0.1 mg). The water absorption curves correspond to the average water uptake of the neat structures compared to the 1% wt. GNP-enhanced structures (Figure 5). In all structures, despite the fibre orientation, the GNP-enhanced composites outperformed the neat composites. All coupons exhibited near-identical water uptake curves, with the fibre orientation that presented the highest water absorption being the 90° coupons, despite the resin type, as presented in Table 2. The detrimental effects of water absorption were less evident in the case of the GNP composites, leading to higher retention of mechanical properties compared to their neat counterparts.

Due to the natural affinity of epoxies to absorb moisture, considerable research efforts have been made to reduce that effect. Epoxies absorb water within the voids of their polymeric network. Generally, two types of water can be identified when water is absorbed by epoxies:

- Unbound free water (Type-I water), which occupies nano-voids within the epoxy without inducing any significant swelling.
- Hydrogen-bonded water (Type-II water), which is responsible for causing swelling in the epoxy due to the formation of multiple hydrogen bonds with unreacted epoxy groups [25,26].

In the initial absorption stage, water ingress increases linearly with time until it reaches a saturation point. This saturation point is linked to the free volume within the polymer. After reaching the saturation point, stage-2 absorption begins and the rate of water ingress decreases. During this stage, Type-II water molecules form hydrogen bonds with unreacted epoxy groups, leading to swelling of the polymer. This, in turn, results in the degradation of the material’s performance [11]. In GFRP, water absorption can occur with various mechanisms either at the locations of micro-cracks in the matrix or at the interface between the fibres and the matrix. The latter mechanism involves the diffusion of water into the surrounding polymer network through unreacted polymeric chains. In highly cross-linked epoxy systems, the increased free volume tends to result in higher absorption of Type-I water during the early stages of exposure [27]. Consequently, when GNPs are effectively dispersed within the epoxy network, the reduction of available free space within the material can lead to notable enhancements in both the sorption (absorption) properties and the mechanical characteristics of the composite system. The sorption curves for both groups followed the same trend. The GNP-enhanced GFRP had an aggregate water absorption reduction of 12%, which is a significant improvement considering the already low absorption of the neat coupons. As separate groups, the GNP modification of the GFRP improved the water absorption by as much as 40% in the case of the 90° coupons. The improvement is even higher if the difference in fibre content is considered due to glass fibres’ higher hydrophobicity compared to the epoxy resin. In Figure 5 it is observed that despite fibre orientation the water uptake curves have similar trends, with most of the water intake taking place in the first sec^1/2^ of exposure. These trends can be attributed to coupon similarities since the core materials are the same, manufactured with the same method, and weighed on the same day during exposure in the hydrothermal chamber. Their only difference is their edges, which were sealed with commercial silicon.

### 3.2. Physical and Mechanical Characterization

**Figure 6:**
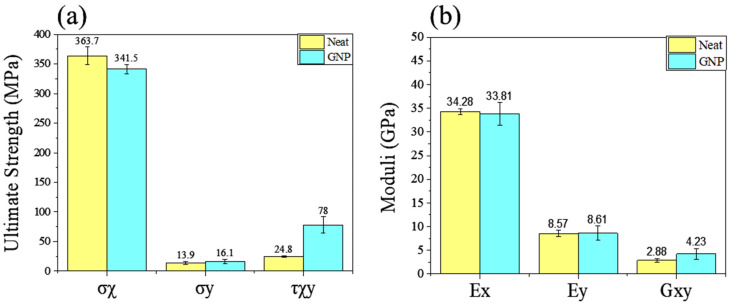
Pristine coupons (**a**) strength and (**b**) moduli. Ex and σx are the Young’s modulus and ultimate strength of the 0° coupons; Ey and σy for the 90° coupons. Gxy and τxy are the in-plane shear moduli and strengths of the ±45° coupons.

The coupons that incorporated nano-modifications with fibre orientation parallel to the loading direction displayed an ultimate tensile strength that was 7% lower than that of the unmodified coupons and near-identical Young’s modulus as seen in Figure 6. This observed behaviour can be primarily attributed to the reduction in fibre content compared to the unmodified GFRP. The tensile strength of 0 oriented composite materials is mostly affected by the fibre content. The ultimate tensile strength of the fibres is significantly higher than the tensile strength of the matrix and, as a result, the longitudinal tensile strength is mostly affected by the volume of fibres in the system. In composite coupons oriented at θ = 90°, failure is primarily due to the occurrence of transverse matrix cracking. While one might intuitively assume that in these coupons where the fibres are not under tensile stress the material would exhibit the characteristics of a pure polymer, the presence of transverse fibres has an adverse impact on the tensile strength of the coupons caused by debonding between the fibre/matrix interface. This debonding phenomenon leads to a reduction in tensile strength perpendicular to the orientation of the fibres [28]. Compared to the neat coupons, nanomodified coupons showcased an increase in tensile strength perpendicular to the fibre orientation of over 10%, while the modulus of elasticity was relatively the same. An impressive increase due to the nano-modification of the resin was exhibited by the coupons subjected to in-plane shear according to ASTM—D3518. The nano-modified coupons exhibited ultimate shear strength over 200% compared to the neat GFRP. Carbon nanofillers show an improvement in the shear strength of composites. Pinto et al. observed an increase in shear strength of more than 50% with incorporation of 0.1 wt.% GNPs [25]. The effective reinforcement of the epoxy matrix can be attributed to the 3D orientation of GNPs. GNPs introduce additional reinforcement mechanisms, such as bridging effects, enhancing mechanical interactions between the fibres and the matrix and, as a result, increasing shear strength [29].

**Figure 7:**
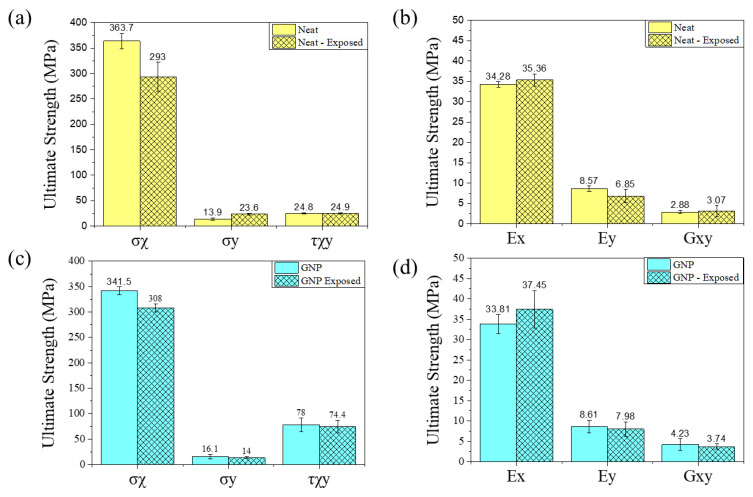
Moduli and strength comparison of pristine and aged (exposed) coupons. (**a**) Strength of neat coupons, (**b**) moduli of neat coupons, (**c**) strength of GNP coupons, (**d**) moduli of GNP coupons.

Environmental aging had a notable impact on the mechanical properties of GFRP coupons as observed in Figure 7. In the case of coupons oriented at 0°, both systems showed an increase in their Young’s modulus. Neat GFRP exhibited a 3% increase in Young’s modulus after aging, while GNP-GFRP exhibited a significant increase of 10%. However, the neat coupons experienced a 20% drop in ultimate tensile strength (UTS), whereas GNP-GFRP demonstrated better retention of mechanical properties with a 10% decrease, showcasing higher UTS post environmental degradation despite the lower fibre content.

For the 90° coupons, neat coupons appeared to show an almost 70% increase in UTS. This apparent increase can be attributed to curing reactions occurring during environmental exposure, often referred to as “pseudo cross-linking”, which results in improved properties [29]. Despite the increase in UTS, the Young’s modulus decreased by 20%. Regarding the GNP 90° coupons, both the Young’s modulus and the UTS presented a drop of 7% and 13%, respectively. For the ±45° coupons, the in-plane shear strength remained relatively stable and did not exhibit significant changes.

### 3.3. Dynamic Mechanical Analysis

**Figure 8:**
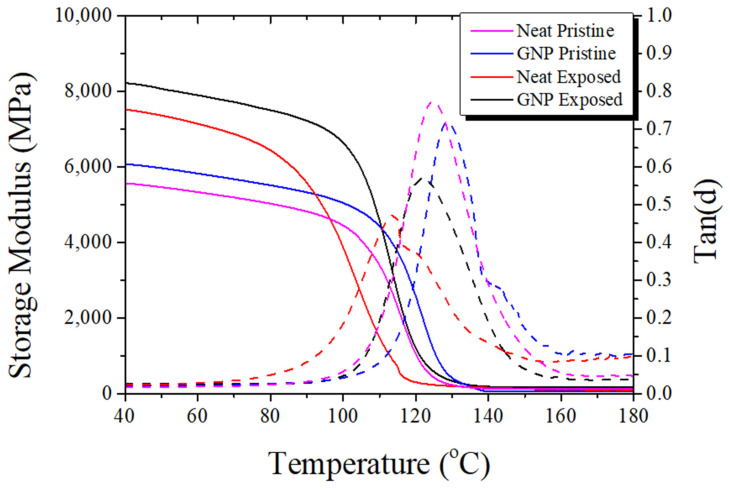
Storage modulus (MPa) and tan (d) of the measured pristine and environmentally aged (exposed) coupons.

In pristine coupons (those without exposure to hydrothermal conditions), the incorporation of GNPs into the epoxy increased its storage modulus by nearly 10% compared to the pure epoxy (Figure 8, Table 3). This enhancement can be attributed to the stiff nature of GNPs, which restrict the movement of polymer chains, despite the smaller crosslink density indicated by the molecular weight between crosslinks’ (Mc) values. Similarly, the inclusion of GNPs led to an increase in the glass transition temperature (Tg) of the material. This rise in Tg is due to the strong interaction between the stiff GNPs and the epoxy matrix, reducing both the mobility of the polymeric chains and the free volume [30]. Despite the reduction in the crosslink density of the material mentioned above, the GNP sample experiences higher thermal stability compared to the neat resin, reaching the rubbery state at higher temperatures [31]. As expected, the addition of GNPs in the material also led to a decrease in the height of the tan (d) curve, indicating higher energy dissipation compared to its internal losses. The tan (d) curve of the neat coupons peaked at 0.77 compared to the 0.71 of the GNP coupons. The decrease in the half-width of the tan (d) curve is noticeable, which can be interpreted as a decrease in the heterogeneity of the polymeric network and lower distribution of the relaxation times of the polymer chains compared to the neat GFRP [32].

When comparing the pristine coupons to those that have undergone hydrothermal aging, a significant increase in the storage modulus is evident: both the pure epoxy and GNP-enhanced resins showed an increase of more than 20%. The storage modulus of the pure resin increased from 5564 MPa to 7522 MPa at 40 °C, while the GNP-enhanced resin increased from 6074 MPa to 8222 MPa. During hydrothermal aging, different mechanisms come into play. The prolonged exposure to elevated temperatures and moisture can lead to secondary post-curing mechanisms, increasing the crosslink density of the coupons. It has been reported that when epoxies are immersed in water, pseudo cross-linking phenomena can occur. Water molecules, both in the free volume of the resin and the water bound to the polymer chains, restrict the mobility of polymer chains, leading to increased stiffness and binding with unreacted polymer chains [11]. This notion is supported by the lower Mc values of the aged coupons, with the neat coupons’ Mc decreasing from 156 to 146 g/mol and the GNP-enhanced coupons experiencing a massive decrease of 321 to 112 g/mol.

Hydrothermal aging also significantly affected the behaviour of the coupons. Both types of GFRP showcased a lower tan(δ) peak, consistent with the increased stiffness mentioned earlier. The pure resin, however, exhibited a deterioration in its tan(δ) profile, with a broader peak, indicating increased network heterogeneity. Furthermore, the aged pure resin displayed a distinct leftward shift in its tan(δ) curve and the appearance of a double peak at 113 °C and 119 °C. This corresponds to a drop in Tg of 11 °C compared to the GNP resin’s drop of 7 °C. The GNP coupon’s smaller change in its tan(δ) profile, if connected with the results of the absorption curve, can be attributed to the reduced hydrolytic degradation process in the polymer due to the lower amount of water absorbed into the body of the resin [33]. The GNP-enhanced coupons showcased the lowest tan(δ) peak between all groups due to their enhanced behaviour.

## 4. Conclusions

This study highlights the considerable potential of GNP modifications in reducing moisture absorption and enhancing the mechanical and dynamic properties of composite materials for large structures. The findings revealed that GNP-enhanced composites consistently outperformed their neat counterparts in terms of moisture absorption. The improvement was particularly significant, with up to a 40% reduction in water absorption observed, in the 90° GNP-enhanced coupons.

Moving on to the physical and mechanical characterization of the composite materials, this study revealed some intriguing insights. Coupons with GNP modifications and fibres oriented parallel to the loading direction exhibited a slightly lower ultimate tensile strength (UTS) compared to the neat composites, despite the 5% reduction in fibre content in the GNP-modified coupons. In contrast, GNP-modified coupons displayed a remarkable increase in shear strength, surpassing their neat counterparts by 200%. This study also conducted dynamic mechanical analysis on the pristine and hydrothermally aged coupons, revealing that the incorporation of GNPs into the epoxy resulted in an approximately 10% increase in storage modulus, indicating greater stiffness. Comparing pristine and aged coupons, both the pure GFRP and GNP-enhanced GFRP demonstrated a significant increase of more than 20% in storage modulus post environmental degradation. This increase was attributed to the impact of prolonged exposure to elevated temperatures and moisture, leading to secondary post-curing mechanisms and an increased crosslink density. As a result, the higher retention of mechanical properties and decreased water absorption, with capabilities for mass production, can lead to improved GFRP composites for wind turbine applications by increasing their lifetime with a marginal increase in their cost.

## Figures and Tables

**Table 1:** Nomenclature and physical properties of each group of coupons.

Name	Resin Type	Fibre Orientation (°)	Fibre Content (%)	Density (kg/m^3^)
N_0	Neat	0	72	1874
N_90	Neat	90	71	1793
N_45	Neat	±45	69	1787
MD_0	1% wt. GNP	0	64	1781
MD_90	1% wt. GNP	90	64	1774
MD_45	1% wt. GNP	±45	68	1820

**Table 2:** Water uptake of each group at day 90.

	Water Uptake (%)		
Orientation	0°	90°	±45°
**Neat**	0.22 ± 0.01	0.28 ± 0.01	0.22 ± 0.02
**GNP**	0.16 ± 0.02	0.20 ± 0.01	0.17 ± 0.01

**Table 3:** DMA values for pristine and aged composites.

	Neat Pristine	Neat Exposed	GNP Pristine	GNP Exposed
Tg (from tan(δ) peak)	124 ± 0.55	113 ± 1.8	128 ± 0.05	121 ± 0.50
Mc (g/mol)	159 ± 70	146 ± 59	321 ± 30.5	112 ± 22.5
tan(δ)peak	0.77 ± 0.05	0.47 ± 0.01	0.71 ± 0.01	0.56 ± 0.01
Width tan(δ) at half peak maximum	22.7 ± 0.93	26.8 ± 3.05	19.0 ± 1.30	22.8 ± 1.05

## Data Availability

Data are contained within the article.
